# Oral Alterations in Heritable Epidermolysis Bullosa: A Clinical Study and Literature Review

**DOI:** 10.1155/2022/6493156

**Published:** 2022-05-31

**Authors:** Alessandro Polizzi, Simona Santonocito, Romeo Patini, Vincenzo Quinzi, Stefano Mummolo, Rosalia Leonardi, Alberto Bianchi, Gaetano Isola

**Affiliations:** ^1^Department of General Surgery and Surgical-Medical Specialties, School of Dentistry, University of Catania, AOU “Policlinico-San Marco”, Via S. Sofia 78, 95124 Catania, Italy; ^2^Department of Life, Health, & Environmental Sciences, Postgraduate School of Orthodontics, University of L'Aquila, P.le Salvatore Tommasi 1, Ed. Delta 6, 67100 L'Aquila, Italy; ^3^Department of Head, Neck And Sense Organs, Fondazione Policlinico Universitario Agostino Gemelli, Università Cattolica del Sacro Cuore, Largo Agostino Gemelli, 8, 00168 Rome, Italy

## Abstract

Epidermolysis bullosa (EB) is a group of skin disorders with skin fragility characterized by blistering from minimal mechanical trauma with rupture at the dermoepidermal junction. There are four major classical heritable EB types, due to mutations in as many as 20 distinct genes: EB simplex (EBS), junctional EB (JEB), dystrophic EB (DEB), and Kindler EB (KEB). This study is aimed at reporting case series on patients (*N* = 8; males, *n* = 5 and females, *n* = 3, age range 12-68 years) affected by EB and performs a review of the literature on this topic. This group of disorders can affect oral soft and hard tissues in various ways, resulting in various effects including enamel hypoplasia, dental caries, microstomia, ankyloglossia, oral blistering, and ulcerations early-onset periodontal disease. From the sample results, it can be concluded that the clinical manifestation of EB patients is highly variable and very different in prognosis. Oral health deeply influences the quality of life of EB patients. Dental management is essential to prevent the aggravation of soft tissue damage and tooth loss and to improve the quality of life through prosthetic and restorative therapies. Dentists should consider the oral alterations of EB subtypes to perform a personalized approach to the patients' needs in a preventive and therapeutic point of view.

## 1. Introduction

Epidermolysis bullosa (EB) is a group of skin disorders with skin fragility characterized by blistering from minimal mechanical trauma with rupture at the dermoepidermal junction [1], leading to erosions and nonhealing ulcers, and, in certain subtypes, can be associated with mutilating scarring and early development of aggressive squamous cell carcinomas [2]. A recent consensus reclassification leads to the identification of four major classical heritable EB types due to mutations in as many as 20 distinct genes: EB simplex (EBS), junctional EB (JEB), dystrophic EB (DEB), and Kindler EB (KEB) [3]. EBS is characterized by alterations of the epidermal basal layer, whereas JEB shows modifications in the lucida lamina and DEB in the dense sublamina [4]. Other heritable “EB-related” disorders with skin fragility are classified as separate categories since blisters are not a predominant manifestation because skin cleavage is very superficial: these includes peeling skin disorders, erosive disorders, hyperkeratotic disorders, and connective tissue disorders with skin fragility [3]. Another variant is epidermolysis bullosa acquisita (EBA), an autoimmune disease with recalcitrant blisters of the skin and mucous membranes induced by autoantibodies to type VII collagen, a constituent of anchoring fibrils of the dermal-epidermal junction [5, 6]. There is no definitive cure for this devastating group of disorders, but novel therapeutic options are under development [2, 5].

The systemic and oral tissue alterations vary greatly based on EB subtype. Regarding oral soft tissues, in mild EB subtypes, the patients may show only occasional vesiculobullous lesions with rapid healing and without scarring: in these cases, the patient's life is not significantly altered. On the other hand, in more severe cases, the entire oral mucosa may be affected by severe blistering with scarring and subsequent microstomia, vestibule obliteration, tongue denudation, and ankyloglossia. These patients have a soft diet and difficulties with mechanical removal of oral biofilm. The dentition may be affected severely by caries due to the presence of dental anomalies such as enamel hypoplasia, depending on the EB subtype [4, 7]. The phenotypes are closely related to the specific abnormal or absent proteins caused by the genetic mutations: for example, type VII collagen (altered in DEB) is essential for skin and oral mucosal integrity and in pathologic conditions, the mutations led to a normal dentition but also a severely affected oral soft tissues. In contrast, laminin 332 (altered in some JEB subtypes) is involved in tooth development; so, a mutated/absent protein led to enamel defects like amelogenesis imperfecta; EBS subjects show mutations in genes (such as *PKP1*,*DSP*, *KRT5*, *KRT14*, *PLEC1*, and *ITGA6*) all causing intraepidermal cleavage; in fact, these patients usually report oral blistering and ulceration [3, 8]. Salivary flow is not affected in EB patients, and the rampant dental caries seen in EB severe forms are likely attributable to nonsalivary factors such as enamel involvement, soft tissue alterations, and/or diet [9].

Given that the clinical management of the EB spectrum disorders is very complex and requires a multidisciplinary approach, the aim of this study is to report a case series showing examples of clinical approaches to these patients and a brief literature review on the main oral alterations in the different forms of EB and on the principles of dental management.

## 2. Case Series

We present a case series of 8 patients affected by mild forms of EB. The subjects, 5 males and 3 females aged between 12 and 68 years, were firstly screened in Oral Medicine Clinic in which dentists and dermatologists collaborate in the same visits. The management of specific treatments (such as dental therapies or skin biopsies) was carried out in the respective specialized clinics.

### 2.1. Case 1

**Figure 1:**
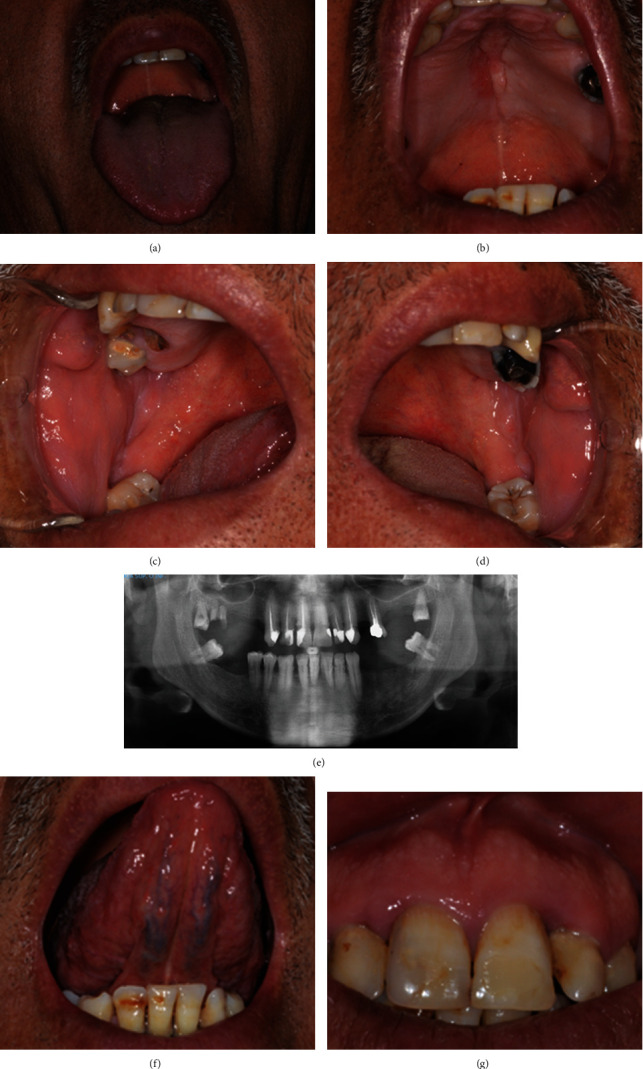
59-year-old nonsmoker man. (a) Tongue. (b) Palate. (c) Right buccal mucosa. (d) Left buccal mucosa. (e) Orthopantomography showing moderate periodontitis associated to alveolar bone loss. (f) Inferior and (g) superior incisors showing minor enamel defects.

A 59-year-old nonsmoker man with JEB presented with erythematous and slightly crusted lesions on the back of the hands and in the retroauricular area. The patient reported an episode of heart attack at the age of 52 managed with acetylsalicylic acid. Intraorally, there was an erythematous lesion on the palate which made its appearance about 1 year ago (Figure 1). Symptoms reported were occasional burning exacerbated with heat and friction. The dentition, partially compromised, showed diffuse and minor enamel defects and some carious lesions. The histological outcome of an incisional periauricular skin biopsy reported the presence of acanthosis, hyperkeratosis, and formation of subepithelial blisters. Palatal erosion was treated with 0.05% clobetasol gel two applications per day and 0.2% hyaluronic acid gel, one application per day, for 3 weeks. The therapy led to a reduction in the extent of the eroded area. One tooth needed extraction (17) and another dental element (18): a restorative treatment. Local anesthesia was used, and there were no complications related to the surgery and other dental procedures. The patient was instructed to maintain good oral hygiene by brushing and flossing, and six-monthly dental visits were recommended to monitor the condition of the teeth and mucous membranes.

### 2.2. Case 2

A 53-year-old nonsmoker woman with JEB reported scattered erosions on her hands, hips, and knees of moderate severity. The patient did not report a specific period of onset but remembered the alternating presence of these lesions from a young age. The oral mucous membranes were intact. Skin lesions caused the patient burning and modest pain due to friction and movements. Dentition was severely compromised with advanced carious lesions in many teeth and widespread enamel defects. Many teeth have been lost, and the bite was collapsed. A periwound incisional skin biopsy on the right hip showed acantholysis and subepithelial blisters. Numerous extractions were performed (teeth: 14-22-36), and some teeth were restored (21-42); an incongruous crown in an upper molar (16) was replaced. A local anesthesia was used in three different interventions, and there were no complications related to the surgery and other dental procedures. The same recommendations of case 1 were given to the patient.

### 2.3. Case 3

A 30-year-old nonsmoker man with JEB showed erythematous and crusted lesions in the retroauricular area. The patient reported the occasional presence of alternating skin lesions since childhood, while the retroauricular lesions had been present for at least 5 months. Oral mucosal lesions were not present. Skin lesions caused the patient moderate itching and burning. Dentition was severely affected by various carious lesions and enamel defects. Anterior superior teeth and relative guidance were loosed. Incisional periauricular cutaneous biopsy showed hyperkeratosis and subepithelial blisters. Some teeth were extracted (teeth: 16-26), but the majority was restored with restorative/endodontic treatment (teeth: 14-15-17-24-25-37). A local anesthesia was used in four different interventions, and there were no complications related to the surgery treatment and other dental procedures. The same recommendations of case 1 were given to the patient. The prosthetic rehabilitation will be subsequently planned.

### 2.4. Case 4

A 12-year-old nonsmoker boy with JEB reported slight and small erythematous and crusted lesions in the retroauricular area without relevant symptoms for about 4 months. Oral mucous membranes were intact, and the dentition showed slight enamel defects without caries. Incisional periauricular cutaneous biopsy showed the presence of epithelial acanthosis and subepithelial blisters. Scaling and root planning procedures were performed, and the patient's parents were instructed on the importance of maintaining a high standard of oral hygiene and six-monthly dental visits. Furthermore, the patient will be planned for orthodontic treatment.

### 2.5. Case 5

A 68-year-old smoker man with EBS presented widespread erosions in the skin of the back, knees, groin, and armpits. The patient was not affected by other organic pathologies; although, he was being treated for depressive symptoms. As reported by the patient, despite having a sedentary lifestyle, the skin lesions, especially exacerbated in the last 3 months, had an intermittent trend with periods of remission and exacerbation, causing intense discomfort. An incisional skin biopsy performed on the left armpit revealed subepithelial blisters and epithelial acanthosis. He was partially edentulous and had a removable prosthesis. The mucous membranes showed erosive lesions in the labial retrocommissures. The patient was advised to apply 0.05% clobetasol gel and 0.2% hyaluronic acid gel for 3 weeks and instructed to maintain high levels of oral hygiene, follow a low-acid diet, preferably tepid, stop smoking, and undergo frequent check-ups. After 3 weeks of treatment, the lesions showed partial healing with almost resolution of the burning symptoms. On the other hand, he showed no commitment to removing the habit of smoking. In addition, the patient was recommended to replace the prosthesis in order to recover the loss of vertical dimension caused by the consumption of resin related to bruxism.

### 2.6. Case 6

A 36-year-old nonsmoker woman with JEB presented eroded skin lesions in hands and feet for about 2 and a half years. A past incisional skin biopsy on the left hand revealed the presence of subepithelial blistering and hyperkeratosis. Oral mucous membranes were free of erosive lesions. However, the dentition was severely compromised due to widespread carious lesions, enamel defects, and roots requiring extractions. The treatment was divided into several steps: first, the teeth with deep carious lesions (14-15-25-32-45) were treated through composite fillings and, where necessary, endodontic treatment (16-26-46). The roots (18-22-27-38-43-47) were then removed through surgery with local anesthesia. There were no oral complications following these procedures. An additional scaling session and topical application of fluoride were performed on the remaining dental elements. The patient will then be followed up for prosthetic rehabilitation. The same recommendations of case 1 were given to the patient.

### 2.7. Case 7

A 64-year-old smoker woman with a past diagnosis of cutaneous EB (25 years ago) presented eroded buccal mucous membranes bilaterally associated with intense pain and occasional bleeding. According to what the patient reported, the appearance of oral lesions dates back to at least 1 year. The patient was also affected by hypertension and periodontal disease; however, tooth hard tissue defects and caries were not detected. Since the buccal cusps of the upper and lower molars appeared quite pointed, a light grinding of these surfaces was performed, and a custom resin splint was produced to reduce the traumatic impact of the teeth on the cheeks, especially at night. Furthermore, the patient was prescribed to apply 0.05% clobetasol gel and 0.2% hyaluronic acid gel for 3 weeks. The patient was also advised to avoid crunchy foods. After two months of follow-up, the symptoms disappeared, and the lesions have significantly regressed.

### 2.8. Case 8

A 44-year-old smoker man with a past diagnosis of cutaneous EB (18 years ago) complained of intense pain in the left mandibular region due to pulpitis on the tooth 46. Deep carious lesions were found in seven teeth (14-16-17-25-27-36-47) and enamel defects in almost all teeth on intraoral inspection. Patient management was carried out in several steps: first of all, acute pulpitis was managed by endodontic and restorative therapy. In several subsequent sessions, scaling, topical application of fluoride, and restorative therapy of the other elements involved were carried out. Only one nonrestorable tooth needed extraction. A local anesthesia was used, and there were no complications related to the surgery and other dental procedures. The patient was strongly encouraged to improve home oral hygiene and to undergo periodic visits at least twice a year.

## 3. Heritable EB Classification

The classification of heritable EB is more complex, because different clinical phenotypes could develop from the same gene (inherited in an autosomal dominant or recessive manner) and, sometimes, different genes or types of inheritance of the same gene could lead to similar phenotypes [3].

### 3.1. Epidermolysis Bullosa Simplex (EBS)

EBS, the most common subtype, consists of skin blistering with a plane of cleavage within the basal layer of keratinocytes, and it is usually inherited in an autosomal dominant manner. Clinical severity is variable, from minor involvement of the feet to extracutaneous forms with a lethal outcome [3].

### 3.2. Junctional Epidermolysis Bullosa (JEB)

JEB, less common than simplex and dystrophic subtypes, is defined by skin blistering due to deeper cleavage within the lamina lucida of the basement membrane and is inherited in an autosomal recessive manner. The two major subtypes of JEB, severe JEB (previously known as Herlitz JEB) and intermediate JEB (previously known as non-Herlitz JEB), are very different in prognosis [3, 10]. The two main proteins mutated in JEB are collagen-XVII and laminin-332 which are both important components of the oral and skin basement membrane [3].

### 3.3. Dystrophic Epidermolysis Bullosa (DEB)

In DEB, the plane of cleavage is localized just beneath the lamina densa in the most superficial portion of the dermis. The inheritance can be autosomal dominant or recessive (the latter form is usually more severe). The main characteristic of this form of EB is the scarring following blistering, both in the skin and mucous membranes [3]. DEB is the second most common form of EB after EBS and has the most severe presentation in this group of diseases [10]. Type VII collagen, altered in DEB, is present in the basement membrane zone of the oral mucosa and in the early stages of tooth formation [11].

### 3.4. Kindler Syndrome (KEB)

KEB is a very rare form of EB [12], due to a mutation in FERMT1, encoding for Kindlin-1, an intracellular protein of focal adhesions in basal keratinocytes [3, 13].

## 4. Oral Alterations in EB Patients

Oral alterations of EB patients are highly variable and depending on clinical subtypes. These alterations include hard tissues and soft tissues involvement which are summarized in Table 1.

### 4.1. Amelogenesis Imperfecta and Rampant Caries

Amelogenesis imperfecta (AI) is a heterogeneous group of inherited conditions characterized by defective enamel formation (enamel hypoplasia), a condition characterized by autosomal dominant/recessive or X-linked inheritance [14]. All deciduous and permanent teeth are typically affected, with marked variations in clinical phenotypes, depending on the underlying genotypes [15]. The impact of AI on affected patients and their families is considerable [16].

The genes related to AI can be subdivided into 3 large groups. The first group includes genes that, when defective, cause AI alone without further phenotypic impacts, such as *MMP20* [17] and *KLK4* [18].The second group includes genes that, depending on the nature of the pathogenic variant, may cause AI in isolation or as part of a syndrome; an example could be the gene defects related to the laminin 332 (*LM332*) [14]. The last group involves genes in which pathogenic variants always cause AI within a broader clinical phenotype or defined syndrome [19].

Therefore, AI may be a phenotypic enamel manifestation in syndromes like JEB characterized by two major subtypes (severe and intermediate) very different in prognosis [3]. All the genes related to JEB (for example, collagen-XVII and LM332, both important components of the oral and skin basement membrane) are involved in ameloblast cell adhesion critical for enamel normal mineralization [3, 20]. Enamel lesions can vary from pitting to generalized hypoplasia resulting in a very thin layer of tooth enamel; some severe JEB patients also show abnormal tooth eruption [21]. Alterations in LM332 production influence the development of enamel cusp morphology (abnormal presence of multiple cusp-like structures) and prism enamel structure (near the EDJ) in initially secreted enamel [14]. Enamel's deep pits and grooves are related to dental caries; in fact, JEB is characterized by a higher risk of this condition [22].

Many of the genes related to EBS are involved in the odontogenesis in the epithelium of salivary glands, but EBS patients' dentition and salivary flow tend to be normal [9, 23], and there is not a difference in the prevalence of dental caries compared to unaffected populations [22]. However, the reduction in salivary flow seems not to be present in other forms of EB; therefore, it seems plausible that the increase in the prevalence of dental caries in patients with severe forms of EB is attributable to nonsalivary factors, such as enamel composition, dietary habits, and the health of the oral mucous membranes or periodontal biotype [9, 24, 25].

Type VII collagen, altered in individuals with DEB, is not expressed by ameloblasts, and the enamel is normal in these patients, but in severe recessive subtypes, they have a higher prevalence of dental caries due to the necessity of soft diets (frequently high in carbohydrates), slower eating (because of marked oral blistering and ankyloglossia), and the lack of the ability to practice a normal oral hygiene, resulting in a prolongated contact of the dental surfaces with potentially cariogenic substrates [7, 8]. The dentition is not affected in individuals with KEB [8].

### 4.2. Early-Onset Periodontal Disease

Periodontitis is an inflammatory disease with bacterial etiology, of the periodontium which is characterized by a progressive destruction of the soft and hard tissues supporting the tooth, leading to alveolar bone loss, pocket formation and, finally, tooth loss [26].

Recessive DEB is one of the most aggressive variants of EB. These patients usually have serious oral discomfort due to oral blistering and pain and microstomia, causing feeding and oral hygiene difficulties. These factors favor extensive plaque deposits and the onset of severe forms of gingivitis and periodontitis that are not easy to manage both in terms of domiciliary and professional oral hygiene [27, 28]. In fact, gingivitis scores showed to be incremented in DEB patients compared to controls, both in primary and permanent dentition [29]. Increased mobility and alveolar bone loss have been also reported [30].

Kindlin-1, altered in individuals with KEB, is expressed in the epithelium that attaches the oral mucosa to the tooth; so, these patients are at risk for developing a severe early-onset periodontal disease [31].

### 4.3. Oral Soft Tissue Alterations

The mouth can be considered the body's window since it can reflect a state of systemic disease. Most of the oral mucosa embryologically derives from the ectoderm invagination; therefore, it is not surprising that it could be involved in disorders associated primarily with the skin.

A 35% of the localized and 59% of the generalized EBS patients show blistering and ulceration of the oral mucosa, but these lesions tend to be few and small in size (<1 cm) [32]. These lesions are localized, often secondary to trauma, and without scarring, but some EBS subtypes may provoke severe oral blistering and scarring [8].

JEB patients have a high prevalence of mucosal lesions, but mostly without significant scarring; so, the soft tissue mobility remains stable. However, severe JEB patients exhibit a perioral granulation tissue, which often leads to microstomia (reduced oral opening) and lower mobility of the lips and perioral tissues [32].

Dominant DEB is characterized by tissue fragility, usually without blistering, but patients with severe generalized recessive DEB have extremely fragile mucous membranes that are affected by ulcerations that heal with scarring: this leads to a changing in the oral architecture such as ankyloglossia, loss of palatal rugae and lingual papillae, vestibule obliteration, and microstomia [8]. Furthermore, DEB patients have greater risk of developing keratocysts and cutaneous and oral squamous cell carcinomas [7, 8].

KEB patients, during infancy, show severe oral blistering, which diminishes with age [31].

## 5. Dental Management of EB Patients

Patients with milder EB forms do not need particular changes regarding dental treatments. However, each patient must be carefully managed for their significant predisposition to oral tissue lesions. Especially patients suffering from severe forms of EB require special measures to minimize trauma to the soft tissues [33].

In EBS patients, most authors agree that routine dental treatment can be provided with local anesthesia and without difficulties, but the clinicians should, however, carefully ask about their history of mucosal fragility, since manipulation could precipitate oral blistering even in mildly affected patients [7, 34].

An altered approach to oral therapy and anesthetic management is required in individuals with enamel hypoplasia, caries, microstomia, and extreme mucosal fragility (e.g., severe JEB and severe generalized recessive DEB). For example, intraoral local anesthesia should be injected more deeply and slowly in order to reduce the risk of mechanical tissue separation and blistering. A fixed prosthetic rehabilitation is useful for protecting the teeth and restoring the esthetics; removable prostheses can often be tolerated in JEB patients who have lost their dentition [7].

Patients with dominant DEB usually do not require particular modifications to dental treatments; however, periodical dental visits are recommended [32]. Patients with severe recessive DEB with extensive dental caries need fixed dental prostheses such as stainless steel crowns, while removable prostheses and orthodontic treatment are usually contraindicated [7, 32, 35]. In patients with severe oral blistering oral hygiene may best performed with a soft-bristled and small-headed toothbrush. Furthermore, systemic/topical fluorides may help control caries and periodontitis [36–39]. Professional hygiene may be performed gently in severe recessive DEB patients. However, if hemorrhagic bullae appear during/after ultrasonic vibration, the bullae should be drained with a steril needle to avoid their expansion [40].

Patients with KEB should be carefully approached, especially regarding periodontal health, since blistering may occur after dental treatments such as scaling and root planning since these subjects may develop severe periodontitis [41].

A recent systematic review showed that QoL is more affected in women than in men, in children than adults, and in recessive DEB and JEB than in other EB subtypes. Moreover, difficulty in sports, need for bathing assistance, eating difficulties, family relationships, and friendships affecting anxiety and depression, were reported in the participants [42].

In order to improve QoL, it is important to perform multidisciplinary management as early as possible. Dental visits are also frequently recommended, starting (ideally) from the first months of life in cases where an early diagnosis has been made [40]. Two clinical studies reported good results in the management of oral bullae and ulcerations with the use of sucralfate suspension [43] or cord blood platelet gel and low-level laser therapy [44]. However, mouthwashes and oral gels have more commonly been prescribed in addition to these strategies [40]. Regarding adjuvant therapies, alcohol-free chlorhexidine 0.12% may be useful for patients with oral lesions, whereas topical fluoride applications every 3 months have been suggested in EB patients with high caries risk [45].

## 6. Conclusions

Several reviews described the pathophysiology and general management of the epidermolysis bullosa disorders group.

The clinical manifestation of EB patients is highly variable and very different in prognosis. In fact, mutations affecting at least 20 distinct genes have been identified in EB pathogenesis, and this explains the spectrum of phenotypic severity in EB subtypes. Oral health deeply influences QoL of EB patients. Dentists should consider the oral alterations of EB subtypes to perform a personalized approach for the patients' needs in a preventive and therapeutic point of view.

## Figures and Tables

**Table 1:** Principal oral alterations in inherited forms of EB.

Major EB subtype	Variants	Soft tissue alteration	Hard tissue alteration
EBS	Localized and generalized	Possible blistering and ulceration, usually localized and without scarring	—
JEB	Intermediate	Possible blistering and ulceration without scarring	Moderate enamel hypoplasia and risk of rampant caries
	Severe	Possible blistering and ulceration without scarring, perioral granulation tissue, and microstomia	Severe enamel hypoplasia and risk of rampant caries
DEB	Dominant	Tissue fragility, usually without blistering	—
	Recessive	Diffuse ulcerations and scarring: ankyloglossia, microstomia, loss of palatal rugae and lingual papillae, and vestibule obliteration	No enamel alterations but high risk of rampant caries due to soft diet, slow eating and worse oral hygiene
KEB^∗^	—	Sever blistering during infancy which diminishes with age	Risk for severe early-onset periodontal disease

^∗^Rare variants.

## Data Availability

Data of the present manuscript are available by the corresponding authors upon reasonable request.
